# A Rare Case of Primary Follicular Dendritic Cell Sarcoma of the Kidney

**DOI:** 10.7759/cureus.50337

**Published:** 2023-12-11

**Authors:** Amanda E Sion, Josh Palka, Sarah Martin, Rafic Beydoun, Conrad Maitland

**Affiliations:** 1 Urology, Ascension St. John Hospital, Detroit, USA; 2 Urology, Detroit Medical Center, Detroit, USA

**Keywords:** follicular dendritic cell sarcoma, fdcs, extranodal, primary renal, kidney

## Abstract

The objective of this case report is to describe a rare case of primary follicular dendritic cell sarcoma (FDCS) of the kidney. FDCS is a rare soft tissue malignancy that most often presents intranodally with lymphadenopathy of the neck, mediastinum, and axilla. One-third of cases present extranodally and most commonly affect the liver, lung, and tonsils. To date, there have been few reports of retroperitoneal FDCS and, to the best of our knowledge, only two other reported cases with primary renal involvement.

We present a 56-year-old female with end-stage renal disease on hemodialysis who presented to the hospital with a hypertensive emergency. Computed tomography (CT) of the abdomen was obtained revealing a left-sided renal mass and she subsequently underwent left radical nephrectomy.

The pathologic features of the mass revealed oval to spindle cells with eosinophilic cytoplasm, dispersed vesicular chromatin, and small nucleoli found arranged in fascicles, whorls, and storiform patterns with occasional multinucleate forms. The neoplastic cells were immunoreactive to vimentin and expressed cell markers for CD23, CD35, and CD68. These features confirmed a final pathologic diagnosis of primary FDCS of the kidney.

To our knowledge, this is the third case of primary renal FDCS reported in the literature. Extranodal FDCS is rare but does occur and needs to be on the differential diagnosis if pathologic features point to its diagnosis.

## Introduction

Follicular dendritic cell sarcoma (FDCS) is a very rare soft tissue sarcoma first described in 1986 by Monda et al. who reported four cases of non-lymphocytic primary lymph node malignancies. FDCS originates from dendritic retinaculum cells in lymph nodes and is characterized by oval to spindle-shaped cells arranged in nesting, swirling, and storiform patterns [1]. Follicular dendritic cells function as antigen-presenting cells in nodal and extranodal lymphoid follicles and serve an important role in B-cell migration, proliferation, and differentiation. They are a non-migratory population of cells derived from the stroma which additionally provide structural support to the lymphoid follicles [2].

Patients with FDCS present with a slow-growing and painless lymphadenopathy which most commonly involves the lymph nodes of the neck, mediastinum, and axilla. Most cases are asymptomatic, however, patients with abdominal involvement typically present with abdominal pain [2]. Approximately 30% of cases involve extranodal sites such as the liver, tonsils, and intra-abdominal soft tissue. Depending on tumor location, tumor size can range from 1 to 15 cm, with an average of 7.4 cm for extranodal tumors [3].

The mainstay of treatment for FDCS is surgical excision with wide margins. In certain cases, adjuvant chemotherapy regimens typically used for disseminated lymphoma can be useful; however, chemotherapy has shown varying success rates and remains controversial [4]. Prognostic factors include: tumor size (>6 cm), high mitotic count, presence of coagulative necrosis, significant cellular atypia, and nuclear pleomorphisms [5,6]. In this case, we describe the clinical course of a female with primary follicular dendritic cell sarcoma of the kidney and highlight the immunohistochemical findings.

## Case presentation

A 56-year-old female with acute on chronic stage IIIb kidney disease, chronic obstructive pulmonary disease (COPD), and uncontrolled hypertension presented to the emergency department with complaints of nausea, intermittent crampy right lower quadrant abdominal pain, and 2-3 episodes of non-bloody, nonbilious vomiting daily for the past three weeks. The patient reported non-compliance with her blood pressure medications and upon presentation, her blood pressure was found to be 245/191 mmHg with a heart rate and respiratory rate of 113 and 18, respectively. The patient was admitted and initiated on dialysis after lab work was notable for a hemoglobin of 9.2 gm/dL, lactate dehydrogenase (LDH) of 334 units/L, lactic acid of 1.1 mmol/L, potassium of 3.7 mmol/L, creatinine of 18.52 mg/dL, blood urea nitrogen (BUN) of 91 mg/dL and glomerular filtration rate (GFR) of 2 mL/min/1.73 m^2^. The patient later admitted to years of intermittent left flank pain, but denied a history of hematuria, dysuria, or nephrolithiasis.

**Figure 1:**
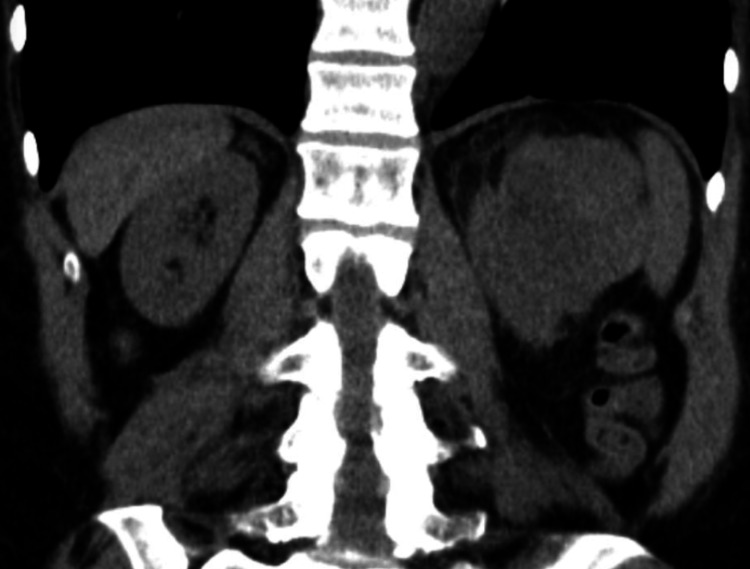
Non-contrast abdominal CT. Lobulated, heterogenous mass arising from the superior posterolateral aspect of the left kidney with focal hyperdense and hypoattenuating areas.

The patients’ blood pressure failed to improve with dialysis and antihypertensives, so a renal ultrasound was performed which revealed a 6.7 x 5.6 x 7.5 cm lobular partially exophytic mass arising from the left kidney with no evidence of hydronephrosis or vascular abnormalities. Due to the patient’s acute kidney injury, a non-contrasted computed tomography (CT) of the abdomen was obtained to further define the extent and nature of the lesion. CT confirmed a lobulated, heterogenous mass arising from the superior posterolateral aspect of the left kidney with focal hyperdense and hypoattenuating areas concerning for hemorrhage and necrosis (Figure 1). There was high suspicion for a renin-secreting tumor and surgical intervention was deemed necessary.

The patient underwent an open radical left nephrectomy approximately one week after admission that was complicated by a grade 1 splenic injury, which was managed conservatively with hemostatic agents. Post-operatively, her blood pressure improved significantly and was well controlled with oral agents alone. She was discharged home on post-operative day 2 and was scheduled for a follow-up with nephrology to continue hemodialysis. The patient did well post-operatively, however, due to her long history of non-compliance she was eventually lost to follow-up.

**Figure 2:**
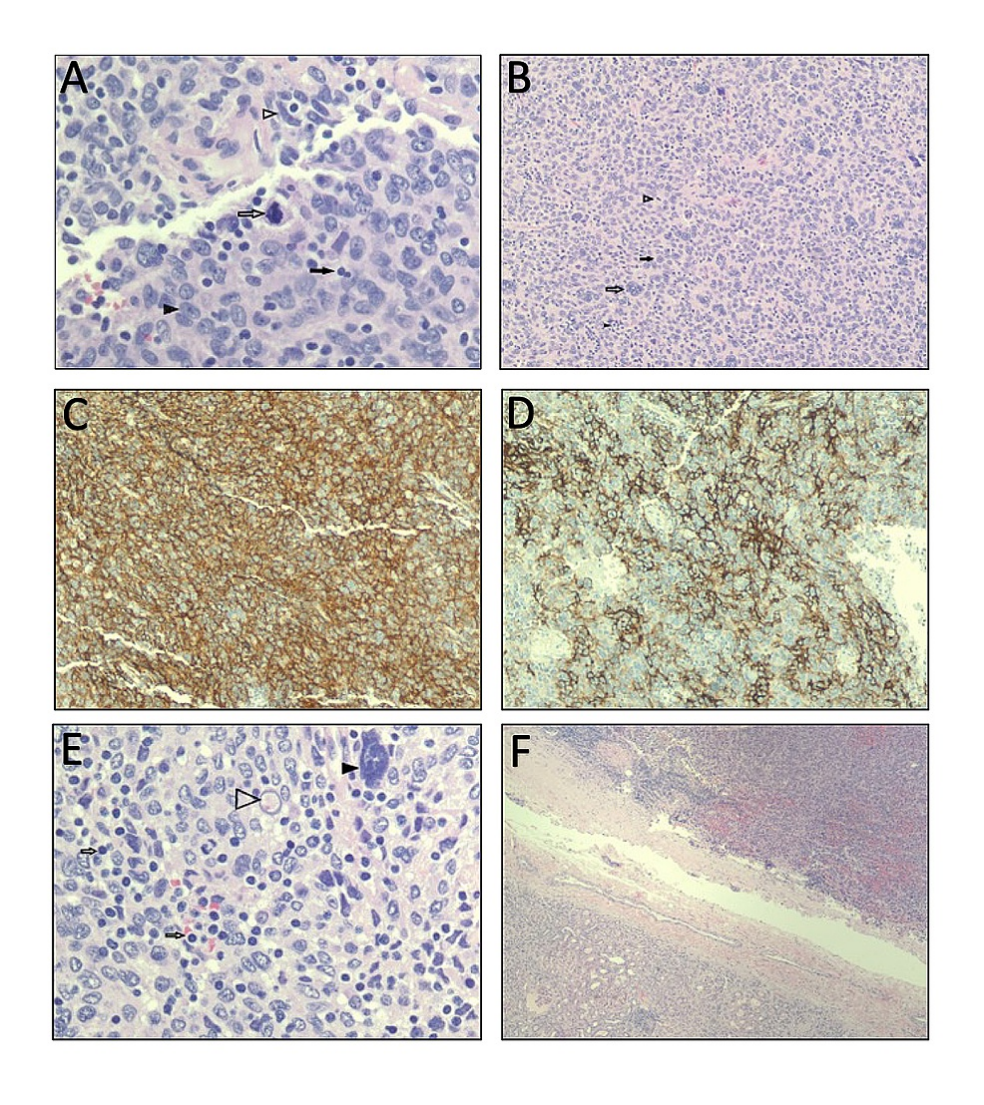
Histological slides of renal tumor. (A) Tumor with oval to spindled cells with dispersed chromatin, binucleated (solid arrow) and multinucleated cells (hollow arrow), inflammatory cells (solid arrowhead), and mitotic figures (hollow arrowhead). (B) Spindle cells (hollow arrowhead) and oval cells (solid arrowhead) with dispersed chromatin, inflammatory cells (solid arrow), and mitotic figures (hollow arrow). (C) Immunohistochemical stains for CD 23 show positivity in the dendritic cells (brown stain). (D) Immunohistochemical stains for CD 35 show positivity in the dendritic cells (brown stain). (E) Tumor with multinucleated cells (solid arrow), inflammatory cells (hollow arrow), and intranuclear pseudoinclusion (hollow arrowhead). (F) Normal kidney (lower left) and tumor (upper right).

Microscopic examination of the surgical specimen revealed a 7.5 cm renal mass confined to the kidney with no extrarenal extension, negative vascular and ureteral margins, and no involvement of the renal pelvis, sinus, or adrenal gland by the tumor. The histologic studies demonstrated a range of architectural features most consistent with FDCS. Oval to spindle cells with an eosinophilic cytoplasm, dispersed vesicular chromatin, and small nucleoli were found arranged in fascicles, whorls and storiform patterns with occasional multinucleate forms. Scattered mitotic figures with some abnormal focal necrosis and perivascular lymphocyte cuffing were found among a mix of both lymphocyte and plasma cells (Figure 2). The neoplastic cells were immunoreactive to vimentin, expressed cell markers for CD23, CD35, and CD68, and stained negative for renin (Table 1). Immunoreactivity for CD21 was not obtained by the pathologist.

**Table 1:** Follicular dendritic cell sarcoma histological immunoreactivity.

CD23	CD35	Vimentin	CD68	CD99	AE1/AE3	HMB45	S100	CD34	CD10	INI-1	TLE1	Desmin	P504
+	+	+	+	+	-	-	-	-	-	-	-	-	-
GATA-3	P63	CA IX	RCC	Bcl-2	PAX-2	PAX-8	CAM5.2	CK7	CK20	CD15	CD30	CK903	ALK
-	-	-	-	-	-	-	-	-	-	-	-	-	-

## Discussion

Retroperitoneal FDCS is an exceedingly rare malignancy and very few cases have been reported in English literature. There have been roughly 343 total cases of FDCS published since the first case reported in 1978, and while the majority of cases involved head and neck lymph nodes, only eight patients to our knowledge demonstrated primary retroperitoneal disease [6,7]. Due to the rarity of the disease, precise risk factors are uncertain; however, FDCS has been associated with Epstein-Barr virus (EBV), autoimmune disease, and follicular cell proliferation in the setting of the hyaline variant of Castleman disease [8,9]. Our patient did not have known preexisting autoimmune or Castleman disease, and cases of FDCS related to EBV predominantly occur in the liver, spleen, or in some cases colon, therefore EBV status was not evaluated.

Pathological diagnosis requires both morphological and immunohistochemical analysis as FDCS displays a wide range of architectural features. FDCS is composed of spindle to ovoid cells arranged in nesting, swirling, or storiform patterns [1,10]. Multinucleated cells can often be seen, as well as an eosinophilic cytoplasm, dispersed chromatin, and small distinct nucleoli [10]. An immunohistochemical analysis should be performed to confirm the diagnosis of FDCS. The markers for follicular dendritic cell origin include CD21, CD23, CD35, and EMA [11,12]. FDCS has variable reactivity for CD68, S100, HLA-DR, and CNA.42, as well as vimentin and desmosomes [10,11]. FDCS consistently stains negative for CD1a, lysozyme, CD34, CD3, HMB45, and cytokeratin [6]. Moreover, an important diagnostic feature of FDCS is the presence of long processes connected by scattered desmosomes in the absence of Birbeck granules [10]. The histological and morphological features of our patient’s surgical specimen had many of the hallmark features of FDCS and a diagnosis of FDCS was made.

In 2019, Lopez-Hisijos et al. reported five cases demonstrating potential pitfalls one can encounter when diagnosing FDCS due to the wide variety of morphological and immunohistochemical features displayed [10]. In particular, Lopez-Hisijos et al. reported a case of misdiagnosed juxtaglomerular cell tumor (JCT) in a 29-year-old female who presented with refractory hypertension which resolved following a nephrectomy similar to our patient. Initially, a diagnosis of FDCS was made due to the sample staining positive for CXCL13, a cytokine produced in B-cells and previously thought to be a reliable marker for FDCS. Given the morphology and immunohistochemical analysis classic for FDCS, after the sample was processed by another institution using electron microscopy and immunohistochemical staining specific for renin granules, the final diagnosis of a JCT was made [10]. This begs the question of whether histological morphology and immunohistochemical staining alone are sufficient to make an accurate diagnosis of FDCS in the kidney. Although hypertension in the setting of a renal tumor is highly suggestive of a JCT, immunochemical markers and morphological features for this condition such as CD34, actin, renin, and renin progranules were negative in our patient [13]. Moreover, there have been no documented connections between hypertension and FDCS. Because our patient’s blood pressure became easily managed following nephrectomy, her hypertension could potentially be attributed to a mass effect on the kidney rather than a paraneoplastic process.

A clinical diagnosis of intranodal or extranodal FDCS is difficult due to the lack of symptoms specific to these conditions. Intranodal FDCS is asymptomatic in nature and may only present with painless lymphadenopathy, while extranodal FDCS most commonly presents with symptoms associated with tumor location and organ involvement. In the literature, retroperitoneal FDCS traditionally does not present with painless lymphadenopathy as does intranodal FDCS. Comparable to neoplasms of the kidney, FDCS is most commonly found incidentally on imaging. For example, one patient with retroperitoneal involvement presented initially with hepatic amyloidosis that was unresponsive to conventional treatments [14], whereas another patient presented with symptoms of diverticulitis or abdominal pain [5,15]. In both of these patients, retroperitoneal FDCS was only found incidentally after CT imaging was obtained (Table 2). These patients appeared to have symptoms related to mass effect and organ involvement rather than lymphadenopathy and systemic symptoms that conventionally accompany intranodal FDCS, except for one patient who presented with fever and weight loss [16]. Similarly, our patient presented with a hypertensive emergency and years of mild intermittent left flank. She was subsequently diagnosed with FDCS only after her hypertensive status and abdominal pain warranted imaging. Interestingly, in one report a retroperitoneal FDCS presenting as a pancreatic head mass revealed high activity corresponding to the mass after a 99mTc-HYNIC-TOC SPECT/CT was performed warranting further investigation into more specific imaging [17].

**Table 2:** Cases of primary retroperitoneal FDCS reported in English literature. FDCS: Follicular dendritic cell sarcoma

Reference	Age (years), sex	Tumor location	Presenting symptoms
Chiaramonte et al., 2001 [14]	38, M	Retroperitoneal, anterior to aorta and posterior to liver	Hepatic amyloidosis refractory to treatment
Androulaki et al., 2006 [16]	33, F	Retroperitoneal, unspecified location	High fever and weight loss
Padilla-Rodriguez et al., 2007 [12]	35, M	Retroperitoneal, displacing bladder	Abdominal mass, no associated symptoms
Purkait et al., 2017 [15]	24, F	Mediastinum, pelvic cavity, and retroperitoneum	Abdominal pain
Bouriga et al., 2018 [6]	48, M	Retroperitoneal, near iliac vessels	Weight loss and epigastralgia
Li et al., 2018 [17]	37, F	Retroperitoneal, pancreatic head	Obstructive jaundice, weight loss, right upper quadrant pain
Jiang et al., 2020 [5]	49, M	Retroperitoneal, celiac axis region	Symptoms consistent with diverticulitis
Lopez-Hisijos et al., 2019 [10]	29, F	Retroperitoneal, right kidney	Hypertension refractory to treatment

The cornerstone of FDCS treatment is wide surgical excision. A retrospective study of 31 patients with FDCS demonstrated no significant difference in five-year overall survival between those receiving chemotherapy or radiation compared with surgery alone [4]. The use of adjuvant therapy is controversial as there are currently no treatment guidelines due to the rarity of the disease [5]. Chemotherapy treatment regimens for lymphomas and sarcomas are thought to be effective for FDCS that are either very bulky or incompletely resected, but more studies need to be conducted to establish a consensus on the most advantageous treatment [6]. Fonseca et al. found that patients responded to cyclophosphamide, vincristine, doxorubicin, and prednisolone (CHOP) or CHOP-like treatments, but recurrence rates remained very high despite achieving a disease-free state. While the durability of chemotherapy regimens for disseminated FDCS is not well studied, consolidative radiotherapy in patients with localized disease that has been either completely or partially resected has been shown to prevent recurrence reliably [18]. Furthermore, a study by Jain et al. established that patients with extranodal, bulky, or intra-abdominal tumors experienced poorer outcomes than patients whose tumors were intranodal and should receive a more intensive treatment regimen [19]. In this report, our patient did not receive chemotherapy following nephrectomy due to the fact that wide negative margins were achieved. There was no extrarenal extension and, vascular and ureteral margins were negative with no involvement of the renal pelvis. The patient was eventually lost to follow-up, and, to the best of our knowledge, the patient has not had a recurrence.

## Conclusions

We present the case of a 56-year-old female who presented with a hypertensive emergency and was incidentally found to have a large renal mass on imaging. FDCS was diagnosed after radical nephrectomy followed by careful morphological and immunohistochemical analysis. To our knowledge, this is the third case of primary renal FDCS reported in the literature highlighting that extranodal FDCS, although rare, does occur and needs to be on the differential diagnosis for pathologists when common morphological and immunohistochemical studies are encountered. While FDCS has variable reactivity for CD68, S100, HLA-DR, CNA.42, vimentin and desmosomes, markers indicative for follicular dendritic cell origin include CD21, CD23, CD35, and EMA.
